# Neural dynamics of emotionally expressive piano performance: a single-case study

**DOI:** 10.3389/fcogn.2026.1945714

**Published:** 2026-10-06

**Authors:** Alice Mado Proverbio, Giulia Carta

**Affiliations:** Cognitive Electrophysiology Laboratory, Department of Psychology, University of Milano-Bicocca, Milan, Italy

**Keywords:** EEG source localization, emotion, hemispheric lateralization, motor expertise, music performance, musicians, swLORETA

## Abstract

**Introduction:**

Understanding how expert performers translate expressive intent into sound remains a central question in the neuroscience of music. We recorded continuous 128-channel EEG from a professional, internationally active concert pianist during a concert-like, uninterrupted performance (~27 min) of six works by Bach, Beethoven, and Chopin.

**Methods:**

From the continuous recording, 99 two-second clean EEG segments were extracted and assigned to one of six previously validated performance-emotion categories based on independent listener evaluations (Joy, Peacefulness, Melancholy, Argumentative, Tension, Power). Each 2-s fragment contained 1,024 time points per channel, yielding 131,072 EEG data points. Cortical and subcortical source generators were reconstructed with standardized weighted Low-Resolution Electromagnetic Tomography (swLORETA), and activation was quantified both as frequency of occurrence and as dipole magnitude across three macro-regions (anterior, posterior, subcortical).

**Results:**

A chi-square test on activation frequencies showed a significant association between cortical/subcortical region and emotional category. Repeated-measures ANOVAs revealed significant region- and emotion-dependent differences in dipole magnitude. Across virtually every emotional category, the left basal ganglia, left thalamus, and left precentral gyrus (BA4) emerged as the most consistently recruited generators, indicating a stable left-lateralized fronto-striato-thalamic axis subserving fine motor control within this performance. Superimposed on this stable motor substrate, emotion-specific modulations emerged: Joy was associated with the highest subcortical activation magnitude, while Melancholy showed the lowest subcortical magnitude together with a selective recruitment of right-hemisphere generators (right BA10, right somatosensory cortex, right insula), consistent with valence-based hemispheric asymmetry accounts. Pearson correlations further linked activation of the left basal ganglia, left thalamus, and left premotor cortex (BA6) to objective performance parameters (gestures per second, notes per phrase, acoustic intensity), suggesting that neurofunctional engagement scales with the motor complexity of the material performed.

**Discussion:**

These findings provide a performer-side perspective complementary to models of music-evoked emotion largely derived from listeners, and suggest that, in this elite performer, expressive intent was instantiated in a specialized, left-dominant sensorimotor-limbic network modulated by affective valence. The study is complemented by animated audiovisual materials that dynamically trace the moment-to-moment evolution of LORETA source activity in synchrony with the unfolding musical passage.

## Graphical Abstract

**Figure:**
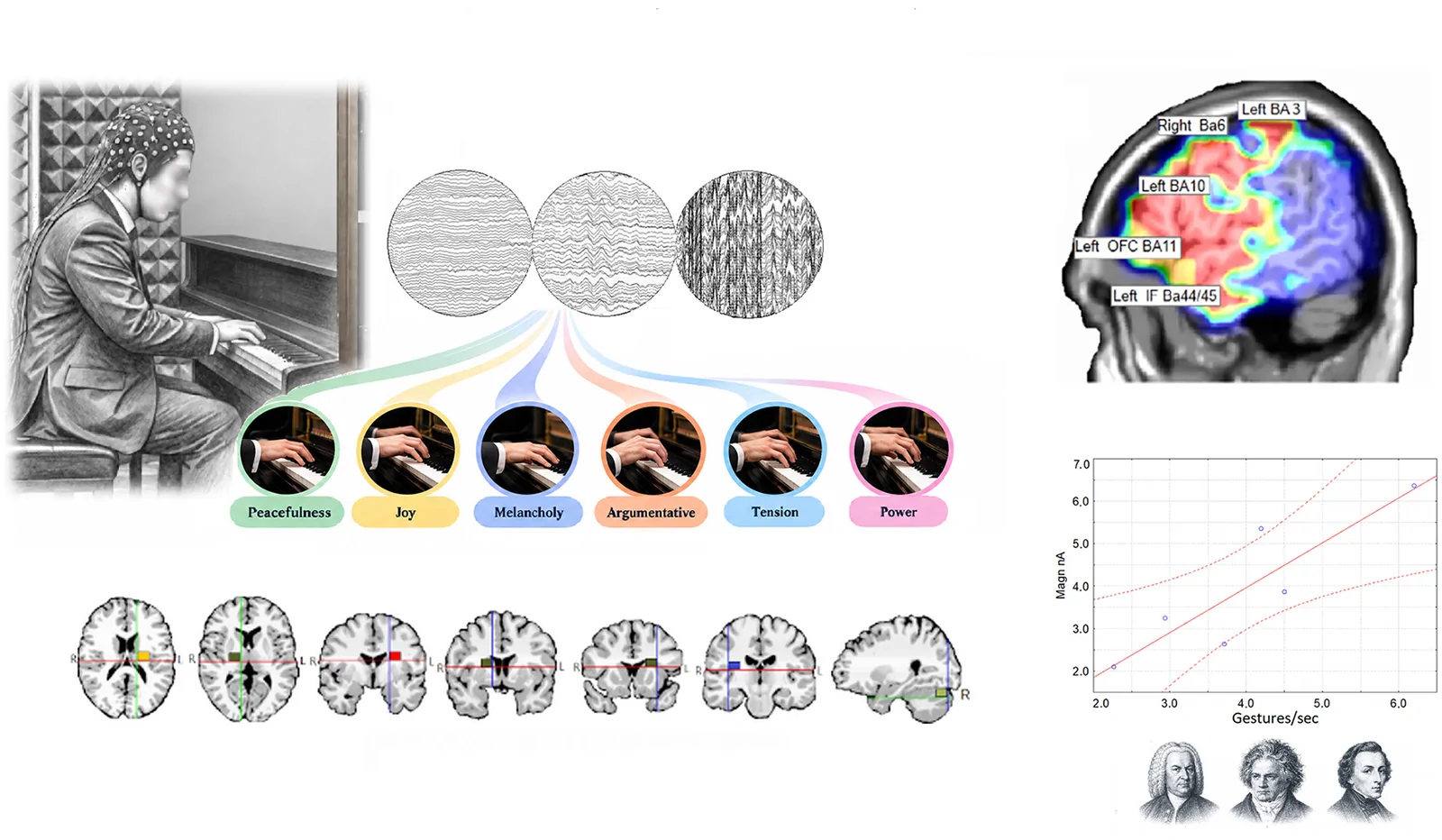

## Highlights

- Continuous high-density EEG (128 channels) was recorded from a world-class concert pianist performing Bach, Beethoven, and Chopin.
- swLORETA source reconstruction mapped cortical and subcortical generators for six validated performance-emotion categories (Joy, Peacefulness, Melancholy, Argumentative, Tension, Power).
- A left fronto-striato-thalamic axis (basal ganglia, thalamus, precentral gyrus BA4) was recruited across virtually all emotional categories, suggesting a consistently recruited motor-executive pattern within this performance.
- Melancholy selectively engaged right-hemisphere structures (BA10, somatosensory cortex, insula) and showed the lowest subcortical magnitude, whereas Joy showed the highest, consistent with valence-dependent hemispheric asymmetry models.
- Neural activation correlated with objective performance parameters, including notes per second, gesture rate, and acoustic energy, linking motor complexity to the degree of neurofunctional engagement.

## 1 Introduction

Music performance offers a uniquely tractable model for studying how the brain integrates cognition, emotion, and fine motor control in real time. While a large body of neuroimaging work has characterized how *listeners* process the emotional content of music (Koelsch, 2014; Vuilleumier and Trost, 2015; Blood and Zatorre, 2001; Salimpoor et al., 2011, 2013; Zatorre and Salimpoor, 2013), comparatively little is known about the neural processes that unfold in the *performer* while an expressive intention is translated into sound. This asymmetry in the literature is notable given that, from a communicative standpoint, the performer is the origin rather than the endpoint of the emotional signal (Juslin and Laukka, 2003; Juslin and Lindström, 2011; Juslin et al., 2013).

A growing line of research on the neurophysiology of piano performance has begun to characterize the motor and auditory-motor networks engaged during skilled playing, including auditory-motor coupling (Zatorre et al., 2007), tempo- and dynamics-dependent muscular synergies (Huang et al., 2024), the timbral consequences of motor variability (Kuromiya et al., 2025), functional reorganization associated with piano training in novices (Olszewska et al., 2024) and in young adults undergoing structured training (Olszewska et al., 2025), and the behavioral structure of pianistic motor sequences (Santisteban et al., 2024). Parsons (2005) used PET imaging to compare professional pianists performing a memorized Bach concerto with the execution of memorized major scales. While both tasks engaged the core sensorimotor network, concerto performance additionally recruited brain regions involved in higher-order motor planning, auditory processing, memory, and emotional integration, including the basal ganglia, thalamus, cerebellum, premotor cortex, and insula. Moreover, concerto performance was associated with stronger deactivation of default mode regions, suggesting enhanced attentional focus and reduced self-referential processing during complex musical performance. Consistent with these findings, Vaquero et al. (2016) demonstrated that long-term piano training is associated with structural plasticity in a distributed network including the putamen, thalamus, hippocampus, amygdala and superior temporal gyrus, highlighting the contribution of subcortical reinforcement-learning circuits and auditory-motor regions to the acquisition and refinement of musical expertise. Overall, the literature converges on the idea that expert keyboard performance recruits a distributed sensorimotor network in which subcortical structures (the basal ganglia and thalamus in particular) play a central role in action selection, timing, and the integration of movement with auditory feedback (Merrick et al., 2023). However, this literature has focused primarily on motor execution as such, largely independent of the emotional content that the performer intends to convey.

A separate and extensive literature has examined how listeners decode emotion from musical structure, showing that acoustic parameters such as tempo, mode, loudness, and note density systematically covary with perceived valence and arousal (Dalla Bella et al., 2001; Gagnon and Peretz, 2003; Bresin and Friberg, 2011; Trochidis and Bigand, 2013; van der Zwaag et al., 2011; Fernández-Sotos et al., 2016). At the neural level, functional imaging studies have associated happy and sad musical excerpts with differential engagement of frontal, temporal, and limbic structures, with a broadly right-lateralized profile for sad, low-arousal material and left-lateralised engagement for positive, high-arousal material (Mitterschiffthaler et al., 2007). In addition, Proverbio et al. (2020a),b) identified partially shared, valence-dependent neural mechanisms for music and affective vocalizations, with negative stimuli preferentially recruiting right temporal regions and positively valenced stimuli engaging pre-dominantly left inferior frontal and temporal cortices (see also Proverbio and Piotti, 2021). This pattern is consistent with classical models of hemispheric asymmetry in emotion, according to which the right hemisphere is preferentially engaged by negative or withdrawal-related affect, whereas left frontal regions are preferentially engaged by positive, approach-related affect (Henriques and Davidson, 1990, 1991; Sutton and Davidson, 1997; Gainotti et al., 1993; Gainotti, 2019, 2021). Whether an analogous asymmetry characterizes the performer's brain, that is, whether the act of expressing a given emotion through movement recruits hemisphere-specific circuits akin to those recruited when perceiving that same emotion, remains an open question that a performer-side design is uniquely positioned to address. Constructionist accounts of musical emotion further caution against expecting rigid, one-to-one neural signatures for discrete emotion categories, emphasizing instead dynamic, context-dependent recruitment of shared affective circuits (Cespedes-Guevara and Eerola, 2018), a view compatible with proposals of partially shared neural mechanisms for the emotional processing of voice, speech, and music (Proverbio and Piotti, 2021; Proverbio et al., 2020a,b; Proverbio, 2025). In this vein, musical performance has increasingly been conceptualized as an embodied, affective, and exploratory practice in which interpretation and improvisation function not merely as reproductive acts, but as generative processes of artistic and experiential knowledge (De Assis, 2018; Stover, 2017).

Source-localization approaches based on swLORETA (Palmero-Soler et al., 2007; Pascual-Marqui, 2002) offer a means of reconstructing the cortical and subcortical generators of scalp-recorded EEG with a favorable trade-off between temporal resolution and spatial specificity, making this technique well suited to characterizing rapid, naturalistic behaviors such as live musical performance, for which fMRI is impractical. In the present study, we recorded continuous 128-channel EEG from a professional, internationally active concert pianist during a continuous performance of works by J.S. Bach, L.v. Beethoven, and F. Chopin, spanning the Baroque, Classical/early-Romantic, and Romantic periods. Short excerpts of the performance were subsequently mapped, on the basis of a companion behavioral validation study (Proverbio et al., 2026), onto six operationally defined performance-emotion categories, or expressive profiles (namely; Peacefulness, Joy, Melancholy, Argumentative/Proactive, Tension, and Power) that partially overlap with, but extend beyond, the classic circumplex model of affect (Russell, 1980) to capture rhetorical nuances specific to musical expression. Here we used swLORETA to reconstruct the cortical and subcortical sources of the EEG signal associated with each excerpt and examined (i) which brain regions were most consistently recruited across the six emotional categories, (ii) whether activation frequency and magnitude differed systematically as a function of emotional category in three macro-anatomical regions (anterior, posterior, subcortical), and (iii) whether the degree of cortical and subcortical engagement covaried with objective structural parameters of the performance itself (acoustic intensity, note density, gestural rate, and manual laterality). Based on the auditory-motor and motor-expertise literature reviewed above, we hypothesized a stable, left-lateralized fronto-striato-thalamic substrate for the motor implementation of expressive performance across emotional categories. Based on hemispheric asymmetry models of affect, we further hypothesized that positively valenced, high-arousal categories (Joy) would be associated with greater left-hemisphere and subcortical engagement than negatively valenced, low-arousal categories (Melancholy), which were expected to show relatively greater right-hemisphere involvement.

## 2 Materials and methods

### 2.1 Participant

The performer is a right-handed, 30-year-old professional male pianist in excellent general health, with no history of neurological or psychiatric disorders, motor or cognitive deficits, and normal audition and vision. He had begun musical training at age four and had accumulated many years of international concert experience, including performances at major concert venues in New York, Nagoya, and Hong Kong. This profile was selected to probe the upper bound of expressive-motor integration achievable in professional pianism. Written informed consent was obtained prior to participation, and the study was conducted in accordance with the Declaration of Helsinki and approved by the Institutional Ethics Committee (CRIP, protocol number RM-2023-605 on 01/10/2023).

### 2.2 . Repertoire and stimulus material

The performer played six compositions in a continuous, uninterrupted performance. The pieces were selected to represent three historical style periods and totalling approximately 27 min: from J.S. Bach's *Die Kunst der Fuge* (BWV 1080), Contrapuncti I, VII, IX, and XI; from Beethoven, the Adagio section (bars 8–114) of the Piano Sonata No. 31 in Ab major, Op. 110; and from Chopin, an excerpt (bars 65–125) from the Ballade No. 1 in G minor, Op. 23. Repertoire selection prioritized rhetorical clarity and harmonic transparency (Bach), formal innovation and emotional depth (late Beethoven), and lyrical, harmonically rich Romantic expressivity (Chopin). Audio was recorded in an anechoic chamber in WAV format, synchronized with the EEG acquisition. The continuous-performance setting was not strictly required merely to establish an association between EEG activity and subsequently assigned expressive categories. It was, however, central to the broader methodological aim of examining expressive musical production under ecologically valid conditions. A trial-based design in which short passages were repeatedly performed under explicit emotional instructions would have substantially altered the phenomenon under investigation by interrupting interpretative continuity and requiring the performer to adopt externally imposed categorical labels such as “Joy” or “Melancholy.” In the present design, by contrast, phrasing, timing, dynamics, motor organization, and expressive shaping unfolded naturally within the continuous musical performance, and the expressive profiles were subsequently identified independently by listeners. Thus, the rationale for the continuous real-time performance was to preserve the natural sensorimotor and interpretative dynamics of expert musical production rather than because expressive intention can only be investigated in a live setting.

### 2.3 Emotional evaluation by listeners

From the continuous performance, 110 excerpts of approximately 10–11 s (“musical phrases”) were initially extracted for behavioral emotion categorization. Following the protocol of the validation study (Proverbio et al., 2026), a sample of 20 professional musicians rated each excerpt, presented in randomized order, on a six-category forced-choice task against a taxonomy of six operationally defined performance-emotion categories: Peacefulness (positive valence, low arousal), Joy (positive valence, high arousal), Melancholy (negative valence, low arousal), Argumentative/Proactive (negative or ambivalent valence, described as constructive/goal-directed drive), Tension (negative valence, high arousal), and Power (high arousal, associated with acoustic energy). These listener-derived labels were intended to operationalize the expressive meaning conveyed by the performance, rather than the pianist's consciously experienced emotion or moment-to-moment categorical intention; accordingly, no retrospective self-categorization by the performer was obtained. Two expert pianists independently validated the correspondence between structural parameters (e.g., minor mode/slow tempo → Melancholy) and emotion labels prior to testing. Classification accuracy was assessed via confusion matrices and per-category precision, recall, and F1 scores, and one-sample t-tests confirmed that mean accuracy for every category exceeded chance level (16.67%; all *p* < 0.001). A subsequent refinement retained the 56 most prototypical excerpts (10 per category, 6 for Peacefulness), improving F1 scores (notably for Melancholy, F1 = 0.71, and Peacefulness, F1 = 0.66). Repeated-measures analyses of variance (ANOVAs) on the acoustic and kinematic parameters of the validated excerpts showed that acoustic energy (dBFS) discriminated arousal level (higher for Power, Tension, and Joy than for Peacefulness and Melancholy), that note density and gestural velocity increased linearly with emotional activation regardless of composer, and that manual laterality varied systematically with arousal, with high-arousal categories more strongly right-hand (left-hemisphere) dominated and low-arousal, introspective categories showing greater left-hand (right-hemisphere) involvement. In particular, Proverbio et al. (2026) demonstrated that specific emotional categories were systematically associated with distinct motor and acoustic features of piano performance (see Table 1). Low-arousal emotions, such as Peacefulness and Melancholy, were characterized by the lowest gesture rate, reduced note density, and the lowest acoustic intensity. In contrast, high-arousal emotions, including Joy, Tension, and Power, were associated with progressively faster execution, greater note density, and higher sound intensity, whereas Argumentative occupied an intermediate position between these two extremes. Within the minor mode, a clear arousal gradient emerged, with motor activity and musical complexity increasing from Melancholy through Argumentative and Tension to Power, while, in the major mode, Joy elicited significantly greater gestural frequency, note density, and acoustic energy than Peacefulness. These findings indicate that emotional expression in piano performance is tightly coupled with measurable variations in movement dynamics and acoustic output, suggesting that the perceived emotional content of music is grounded in systematic motor-acoustic patterns rather than being solely determined by tonal mode.

**Table 1:** Acoustic and performance-related properties of the musical excerpts as a function of emotional category.

Emotion	Notes/phrase	Right-hand notes	Left-hand notes	dBFS	Gestures/sec
Peacefulness	21.60 ± 2.01	19.10 ± 2.44	24.10 ± 2.43	−33.94 ± 0.78	2.94 ± 0.38
Melancholy	30.80 ± 3.29	18.40 ± 2.65	43.20 ± 8.02	−34.77 ± 0.78	2.27 ± 0.16
Argumentative	36.90 ± 1.28	32.60 ± 2.54	41.20 ± 1.65	−28.04 ± 0.78	4.20 ± 0.05
Tension	44.70 ± 3.30	51.50 ± 3.27	37.90 ± 4.24	−23.15 ± 0.78	3.71 ± 0.45
Power	57.00 ± 4.65	64.10 ± 4.98	49.90 ± 4.96	−21.36 ± 0.78	4.50 ± 0.43
Joy	57.75 ± 5.65	63.67 ± 6.58	51.83 ± 5.33	−24.22 ± 1.01	6.20 ± 0.49

Values are reported as means ± standard errors (SE). The table reports the mean number of notes per musical phrase, note counts produced by the right and left hands (per phrase), sound intensity expressed as root-mean-square amplitude in decibels relative to full scale (dBFS), and the number of performance gestures per second.

Notes/phrase *denotes the mean of the right- and left-hand note counts for each excerpt. Taken from Proverbio and Milovanović (**2026**) with the authors' permission. Material protected by CC BY 4.0 license*.

For the EEG source-localization analyses reported here, an initial pool of 145 two-s segments was extracted from the continuous performance. These segments were nested within the longer musical phrases described above and provisionally assigned to one of the six emotion categories on the basis of the behavioral labels. Segments belonging to excerpts that failed to reach 50% classification accuracy (chance = 16.67%) were subsequently excluded, as were temporally adjacent segments whose inclusion could have introduced autocorrelation across continuous musical passages. After applying these pre-defined selection criteria, the final dataset comprised 99 two-s segments, selected to preserve adequate representation across composers and emotional categories. The suitability of these excerpts for subsequent neurophysiological analyses was further supported by a machine-learning study (Proverbio and Milovanović, 2026), in which EEG epochs extracted from the same musical phrases were classified into their corresponding emotional categories using supervised Support Vector Machine (SVM), Random Forest (RF), and Gradient Boosting (GB) algorithms. All models achieved above-chance performance, with the Random Forest classifier providing the highest accuracy, demonstrating that the selected excerpts elicited sufficiently robust and discriminable oscillatory brain activity to permit reliable computational decoding of emotional performance states. These findings provide independent evidence that the musical material employed in the present study generated distinct neural signatures, thereby offering a strong rationale for investigating their underlying cortical generators through LORETA source localization.

### 2.4 EEG recording

EEG was recorded from 128 Ag/AgCl electrodes mounted in high-density Waveguard caps (ANT Neuro) according to the 10–5 international system, online-referenced to algebraically averaged mastoids. Electrode impedances were kept below 5 kΩ throughout acquisition. Continuous EEG was digitized at 512 Hz using the Cognitrace acquisition platform (ANT Neuro) and band-pass filtered between 0.016 and 70 Hz (notch filter = 50 Hz); EOG was recorded concurrently for automatic blink rejection. Offline segmentation and artifact rejection were performed in EEProbe (ANT Neuro). Passages contaminated by excessive noise were marked as bad blocks (e.g., Maidhof et al., 2013) and excluded *a priori*. Movement-related contamination was therefore handled conservatively by exclusion of affected intervals rather than by applying a dedicated movement-artifact correction algorithm; only artifact-free 2-s segments were retained for source analysis. Although the continuous recording spanned the entire performance, subsequent analyses focused on the 2-s segments described above, time-locked to the previously categorized excerpts.

### 2.5 Source localization (swLORETA)

Cortical and subcortical generators of the EEG signal were reconstructed using swLORETA (Palmero-Soler et al., 2007), an extension of sLORETA (Pascual-Marqui, 2002) that incorporates singular-value-decomposition-based lead-field weighting to improve depth accuracy and localization stability under noisy conditions. LORETA serves as a discrete linear solution to the inverse EEG problem, representing the 3D distribution of neural electric activity that maximizes synchronization in terms of orientation and strength between adjacent neuronal populations (represented by adjacent voxels). Both the head model's segmentation and generation were executed using Advanced Neuro Technology, a software program developed by ASA (Zanow and Knösche, 2004). The swLORETA analysis yielded results indicating the statistical activation of a subset comprising 1056 dipoles. These results provide information on both their Magnitude values (in nA) and spatial coordinates following the Talairach and Tournoux (1988) system. A dipole, in this context, represents the potential difference between the basal and apical portions of a cell, resulting in a positive and negative difference with an orthogonal orientation to the magnetic field. Utilizing the Collins dipole table (Collins et al., 1994), each electromagnetic dipole was associated with its respective Gyrus and Brodmann Area.

For each of the 99 segments, active dipoles were classified according to hemisphere, lobe, gyrus, and Brodmann area (BA), and grouped into three macro-anatomical regions for statistical analysis: an *anterior* region (10 areas, including bilateral BA4, BA6, BA10, BA11, and BA44/45), a *posterior* region (11 areas, including bilateral BA1/2/3, BA7, BA19/18, BA20/21, and BA37), and a *subcortical* region (9 areas, including bilateral basal ganglia [caudate/putamen], thalamus, cerebellum, uncus, and insula/claustrum), as detailed in the Supplementary Tables 1, 2). Where multiple Brodmann areas fell within the same macro-anatomical district, the maximum magnitude value was retained. Dipoles not reaching the algorithm's significance criterion (Fisher's F, magnitude ≥ 1) were assigned a conventional floor value of 0.5. A dedicated equivalence table was used to harmonize overlapping anatomical labels across areas belonging to the same Brodmann region. Subcortical source coordinates (Talairach space) were additionally averaged, weighted by activation count, to derive representative centroids for visualization.

### 2.6 Statistical analysis

Two complementary levels of description were used. First, for each emotional category, the *frequency* of activation of each anatomical district (expressed as % of segments in that category showing significant activation) was computed and compared against theoretical expected frequencies using a chi-square test of independence (Area × Emotion), with Cramer's V as a measure of effect size. Second, dipole *magnitude* (in nA) was analyzed by means of three separate repeated-measures ANOVAs, one per macro-region (anterior, posterior, subcortical), with Area and Emotion (6 levels) as within-segment factors. Statistically significant activation was defined according to the Fisher's F-test implemented by the swLORETA software as part of its standard inferential framework for voxel-wise source estimation, using the software-defined threshold of *p* < 0.05. The Greenhouse-Geisser correction was applied whenever the sphericity assumption was violated for factors with more than two levels, and corrected degrees of freedom, ε, and adjusted *p*-values are reported accordingly. Significant main effects and interactions were followed up with Fisher's Least Significant Difference (LSD) *post-hoc* comparisons. Finally, Pearson correlations were computed, separately for the anterior, posterior, and subcortical macro-regions, between (a) activation frequency (%) or magnitude (nA) of each area and (b) five structural performance parameters averaged per emotional category: acoustic energy (dBFS), gestures per second, notes per musical phrase, notes per phrase performed with the left hand, and notes per phrase performed with the right hand. Given their exploratory, hypothesis-generating nature, the correlation analyses were not corrected for multiple comparisons. All analyses were conducted in SPSS, jamovi (v.2.7; The jamovi project, 2025), and *R* (v.4.5; *R* Core Team, 2025).

## 3 Results

Across the 99 analyzed segments, source reconstruction confirmed, as expected given the fine motor demands and functional specialization of expert pianism, a clear pre-dominance of left-hemisphere generators, both in terms of absolute activation frequency in the most heavily recruited areas and in terms of activation magnitude—with the notable exception of the right premotor cortex (implicated in left-hand control and in the holistic planning of agogic expression). Click here to view some animated videos created by sequentially combining six temporally adjacent LORETA solutions and synchronizing them with the corresponding musical phrase performed at each time point. The examples illustrate the neural activity associated with the performance of Chopin's Ballade (peacefulness), Bach's Contrapunctus XI (tension), and the Adagio section of Beethoven's Piano Sonata (power).

### 3.1 Activation frequency by emotional category (chi-square analysis)

The chi-square test on the anatomical-district-by-emotion contingency table was significant, χ^2^(155) = 242.04, *p* < 0.001, although Cramer's V (= 0.22) indicated a small-to-moderate overall association, consistent with a pattern in which a *core* set of regions is shared across emotions while a smaller set of regions is emotion-specific (see Supplementary Table 3). Across allcategories, seven regions emerged as the most consistently recruited generators: (i) the basal ganglia (caudate/putamen), pre-dominantly left-lateralized, active in 85–100% of segments in every category (see Figure 1); (ii) the thalamus (posterolateral and dorsomedial nuclei), pre-dominantly left-lateralized, showing a similarly high and consistent recruitment; (iii) the superior/middle frontal gyrus (BA10), the only region showing near-ubiquitous, often bilateral involvement across allcategories as being involved in attention, control and working memory; (iv) the left precentral gyrus (BA4), recurrently engaged in categories with higher motor demands (Argumentative/Proactive, Tension, Joy); (v) the right premotor cortex (BA6); (vi) the middle/inferior temporal cortex (BA20/21) for music auditory processing, present transversally but with emotion-dependent lateralization (left-dominant for Power and Peacefulness, right-dominant for Melancholy, Argumentative, and Joy); and (vii) the posterior occipito-parietal network (BA18/19: superior occipital gyrus, cuneus, precuneus), particularly engaged during Tension, Peacefulness, and Melancholy, possibly reflecting imagery.

**Figure 1:**
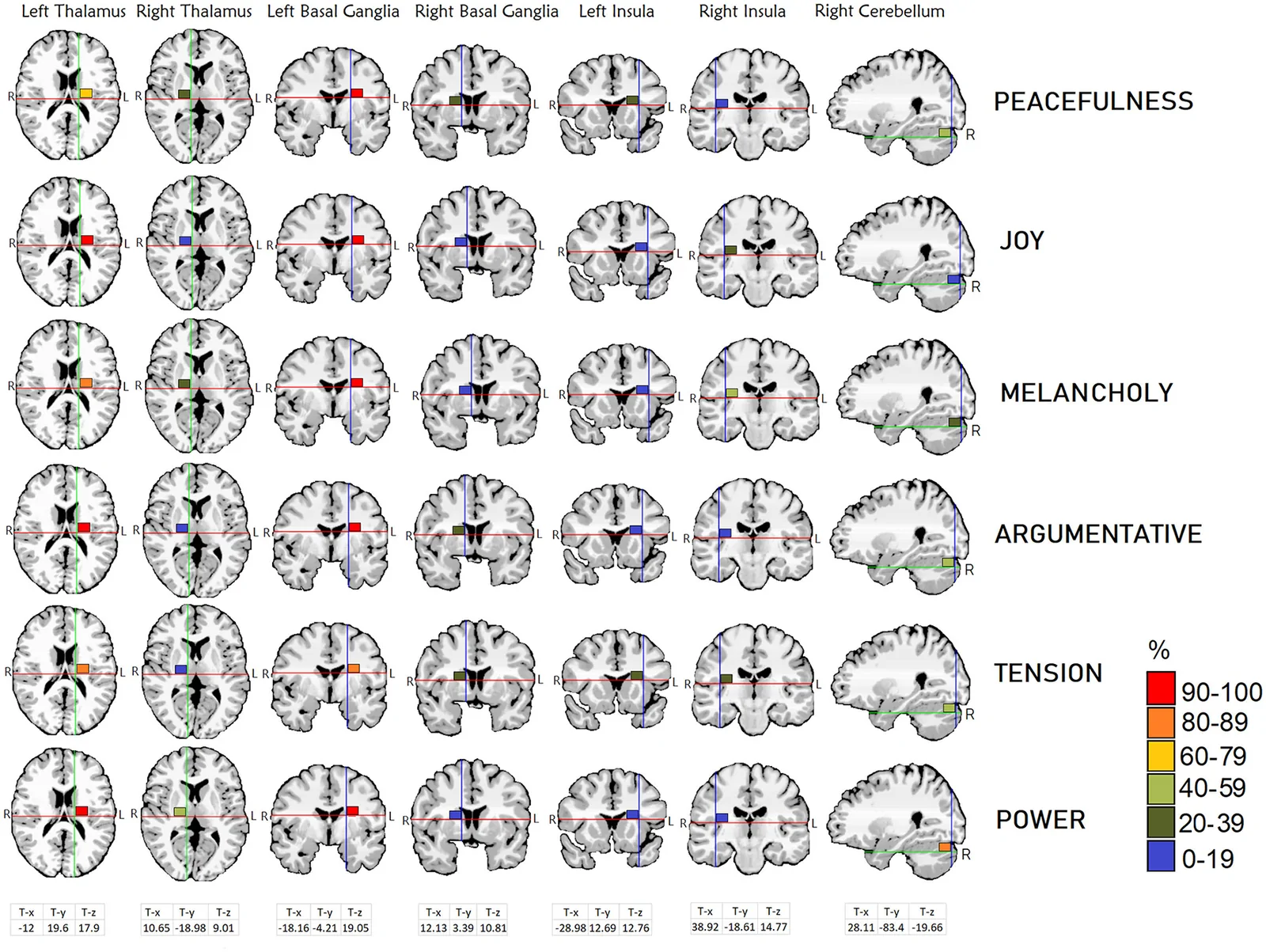
Condition-specific activation frequencies across pre-defined subcortical, insular, and cerebellar regions of interest. Rows correspond to the six affective states examined (peacefulness, joy, melancholy, argumentativeness, tension, and power), whereas columns indicate the anatomical targets: left and right thalamus, left and right basal ganglia, left and right insula, and right cerebellum (left cerebellar activation was relatively infrequent). For each condition–region combination, the colored square marks the stereotaxic target and represents the frequency of activation, expressed as a percentage: 0–19% (blue), 20–39% (dark green), 40–59% (light green), 60–79% (yellow), 80–89% (orange), and 90–100% (red). Crosshairs indicate the location of each target on the anatomical template, and the corresponding MNI coordinates (*x, y, z*) are reported below each column. Left and right hemispheres are indicated in each panel. Source visualization (based on swLORETA Talairach coordinates) was performed using the *Yale BioImage Suite*.

Category-specific profiles are summarized in Figures 1–3. Briefly: *Joy* was characterized by 100% occurrence of the right superior/middle frontal gyrus (BA10) and the left subcortical complex (basal ganglia and thalamus), with strong secondary recruitment (~58%) of the right postcentral gyrus (BA3), left BA4, and left BA10. *Peacefulness* (see Figure 2) showed a comparable subcortical/BA10 core (93.75% left basal ganglia) together with prominent left temporo-occipital involvement (BA19, fusiform gyrus, cuneus, precuneus; 81.25%), and comparatively moderate engagement of motor regions, consistent with an internally guided, reflective performance mode relying on musical imagery and semantic memory rather than direct executive control. *Argumentative/Proactive* excerpts (18/18, 100%) recruited the right BA10 and the left subcortical network together with the left precentral gyrus (88.9%), and showed a right-lateralized prefrontal profile (right BA10 and right BA11) alongside near-total left basal ganglia dominance (right basal ganglia active in only 22.2% of segments). *Tension* was dominated by bilateral BA10 (>90%), strong left subcortical recruitment (basal ganglia 88.9%, thalamus 81.5%), and a left temporo-occipital network (BA19, 70.4%), consistent with sustained visuospatial integration and effort monitoring. *Power* (Figure 3) showed near-total bilateral BA10 recruitment, complete left subcortical engagement, near-total left temporo-occipital involvement (BA19), and, uniquely among the six categories, solid right cerebellar recruitment, suggesting heightened demands on motor error-monitoring. *Melancholy* was the only category showing a marked right-hemisphere signature: right BA10 reached 73.81% (vs. 80.95% left), the right somatosensory/insular complex was comparatively more engaged (right insula/claustrum, 47.62, vs. 19.05% left), while left subcortical recruitment (basal ganglia 90.48%, thalamus 85.71%) remained high, indicating that the core motor-executive substrate persists even for the lowest-arousal, most introspective category.

**Figure 2:**
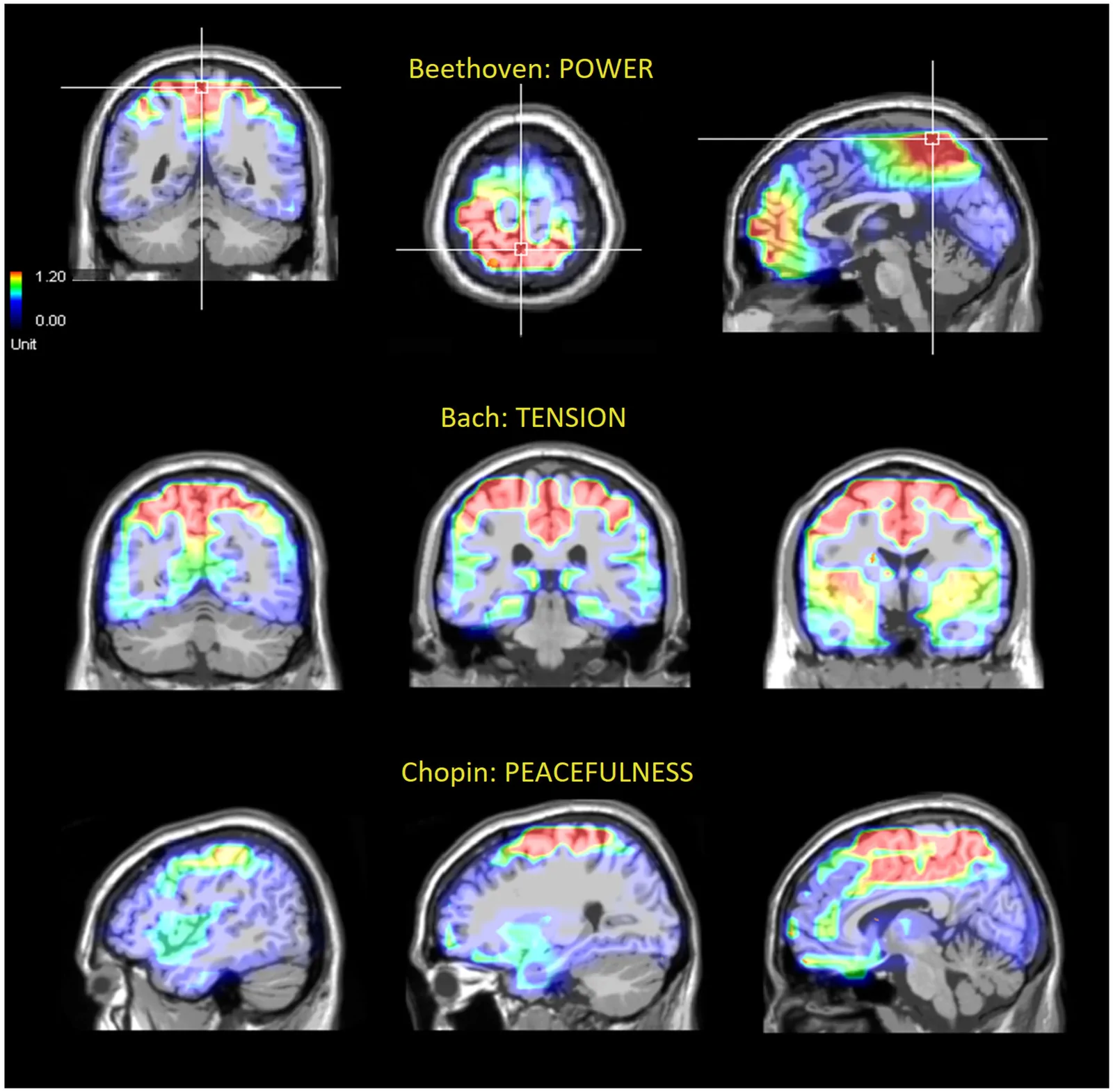
swLORETA source reconstructions for representative 2-s musical excerpts associated with Power (Beethoven, Piano Sonata No. 31 in A-flat major, Op. 110), Tension (J.S. Bach, *The Art of Fugue*, Contrapunctus XI), and Peacefulness (Chopin, Ballade No. 1). Estimated source activity is overlaid on the standard Montreal Neurological Institute (MNI) structural MRI template. Coronal, axial, and sagittal views are shown for Power; coronal sections at different anteroposterior levels are shown for Tension; and sagittal sections at different mediolateral positions are shown for Peacefulness. Source reconstruction was performed using a 5-mm grid spacing and Tikhonov regularization based on an estimated signal-to-noise ratio of 3. The color scale represents the magnitude of the swLORETA current estimates, expressed in nA, with warmer colors indicating greater estimated source activity. Crosshairs indicate the selected source location in the Power reconstruction.

**Figure 3:**
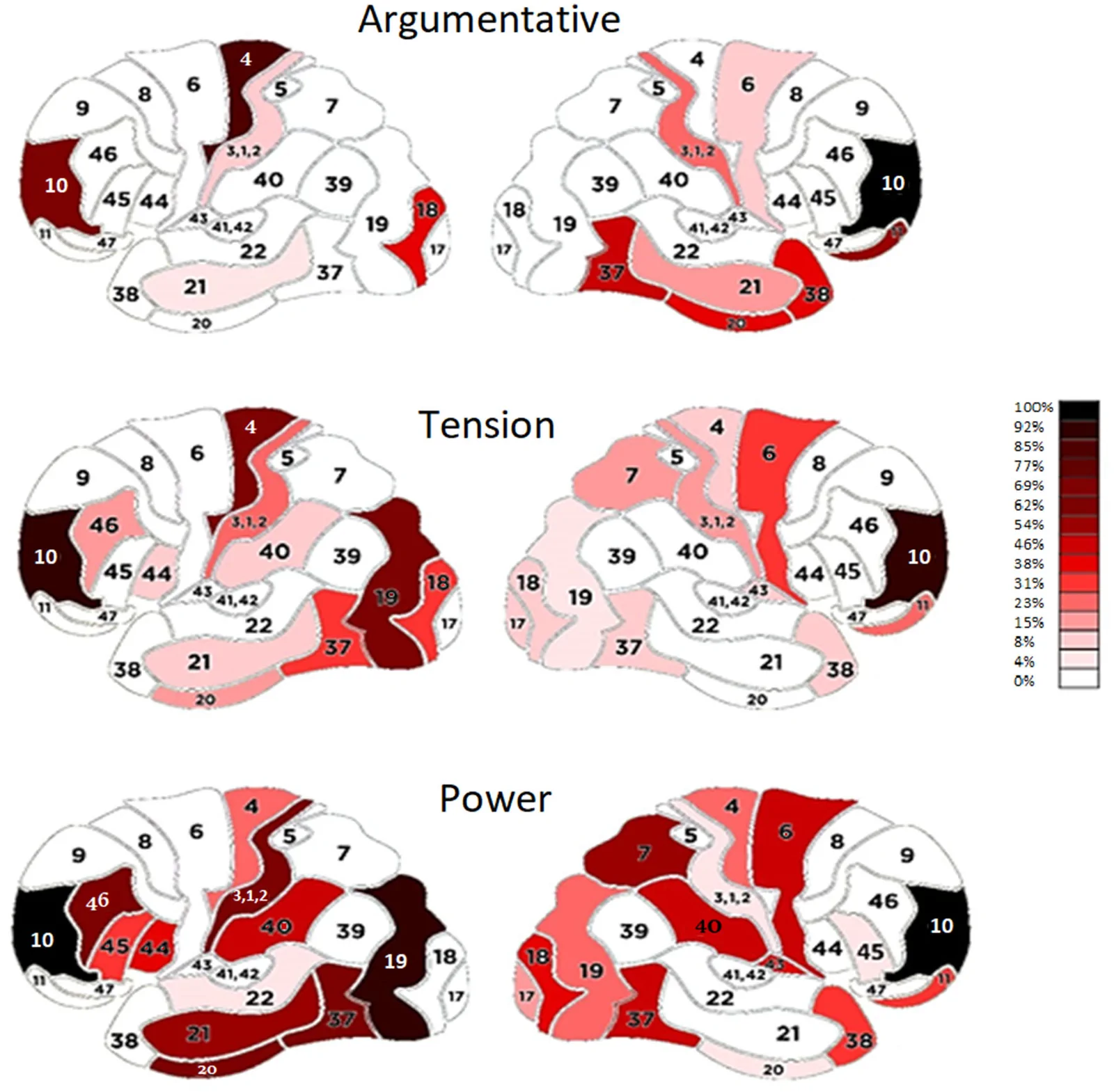
Cortical distribution of activity across Argumentative, Tension, and Power tones. Percentages are based on 99 two-second brain-activity epochs; darker colors indicate more frequent involvement of each Brodmann area. Notably, the number of recruited cortical areas (see for example the right premotor cortex) increased with the mean number of notes per sentence (36.90, 44.70, and 57.00, respectively).

### 3.2 Magnitude analyses (repeated-measures ANOVA)

#### 3.2.1 Subcortical region

The ANOVA showed a significant main effect of Area [*F*_(8, 744)_ = 78.18, *p* < 0.0001 (ε = 0.34, adjusted *p* < 0.0001)], a significant Area × Emotion interaction [*F*_(40, 744)_ = 5.31, *p* < 0.0001 (ε = 0.34, adjusted *p* < 0.0001)], and a significant main effect of Emotion [*F*_(5, 93)_ = 2.36, *p* = 0.046]. *Post-hoc* comparisons on the Emotion main effect showed that Joy elicited the highest subcortical magnitude (*M* = 2.01, SE = 0.26), differing significantly from Tension (*M* = 1.27, SE = 0.20; *p* = 0.027) and, most markedly, from Melancholy (*M* = 1.12, SE = 0.20; *p* = 0.009), which showed the lowest subcortical magnitude overall and differed significantly also from Argumentative/Proactive (*M* = 1.80, SE = 0.21; *p* = 0.024) and Power (*M* = 1.80, SE = 0.25; *p* = 0.040). Peacefulness (*M* = 1.56, SE = 0.23) occupied an intermediate position, not differing significantly from any other category. The Area × Emotion interaction confirmed the pre-dominance of the left thalamus and left basal ganglia as the highest-magnitude generators across nearly the entire emotional spectrum (Figure 4), with the sharpest gradient observed between Joy (left thalamus *M* = 6.59, SE = 0.77; left basal ganglia *M* = 6.37, SE = 0.81) and Melancholy (left thalamus *M* = 2.31, SE = 0.60; left basal ganglia *M* = 2.10, SE = 0.63). Power was further distinguished by increased right cerebellar (*M* = 2.70, SE = 0.36) and right thalamic (*M* = 2.09, SE = 0.30) magnitude, alongside a renewed left thalamic peak (*M* = 4.19, SE = 0.74), not observed for any other category.

**Figure 4:**
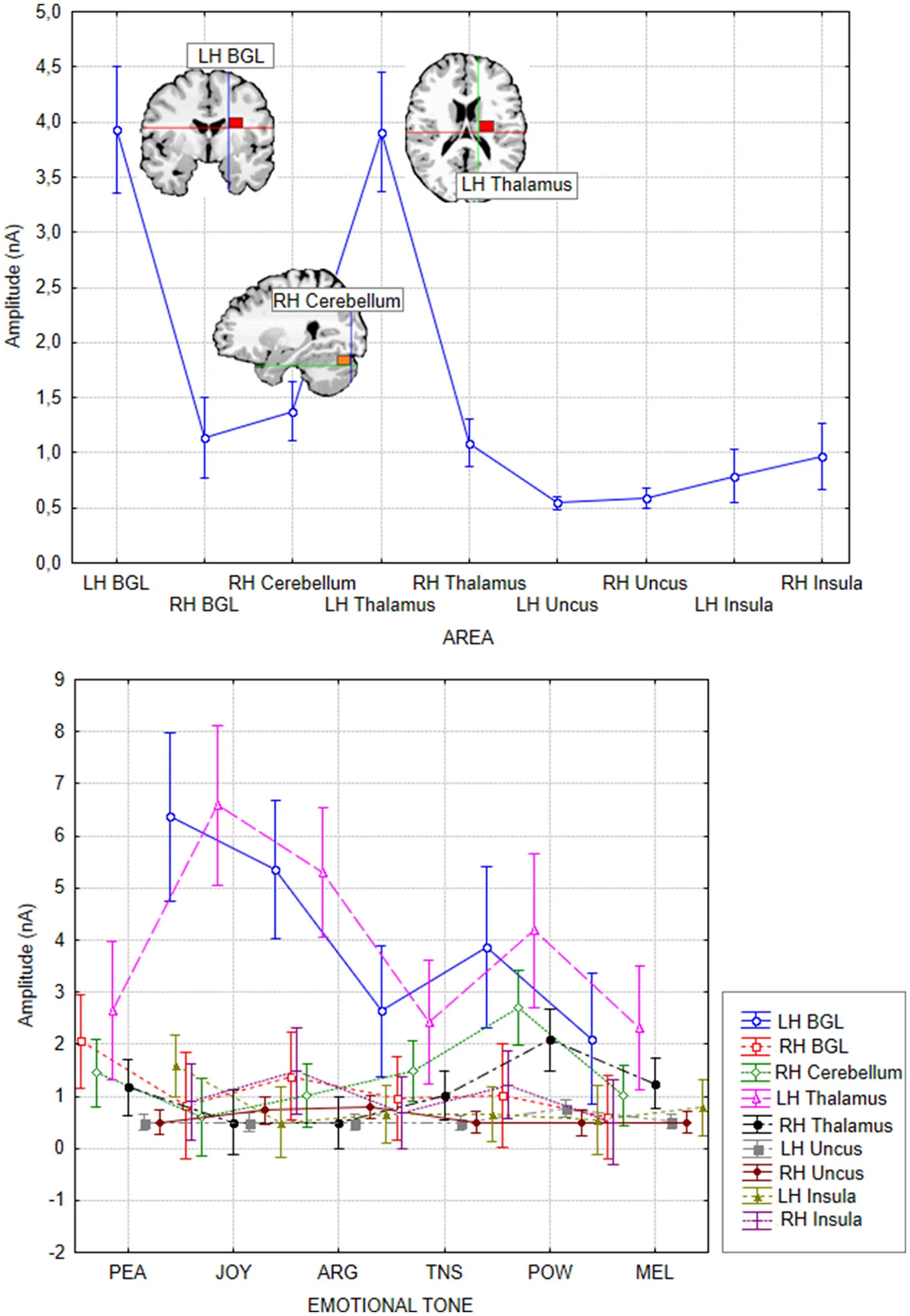
swLORETA-derived source-current amplitudes in subcortical and limbic regions according to repeated-measure ANOVA results. The upper panel shows mean amplitude (nA) in the bilateral basal ganglia (BGL), cerebellum, thalamus, uncus, and insula; anatomical insets indicate representative source locations. The lower panel shows amplitude values across emotional tones (PEA, JOY, ARG, TNS, POW, and MEL). Error bars represent standard deviations.

#### 3.2.2 Anterior region

A significant main effect of Area emerged [*F*_(9, 837)_ = 46.07, *p* < 0.0001 (ε = 0.36, adjusted *p* < 0.0001)], with the highest magnitudes localized to left BA4 (*M* = 4.48, SE = 0.44) and bilateral BA10 (left: *M* = 4.39, SE = 0.37; right: *M* = 4.09, SE = 0.31), as shown in Figure 5, and the lowest to left BA6 (*M* = 0.63, SE = 0.12) and left BA11 (*M* = 0.51, SE = 0.01). *Post-hoc* comparisons revealed a statistically indistinguishable “frontal-motor triangle” comprising left BA10, right BA10, and left BA4 (all pairwise *p* > 0.25), alongside near-perfect bilateral symmetry for BA44/45 (*p* = 0.83). The main effect of Emotion was also significant [*F*_(5, 93)_ = 2.31, *p* = 0.050], driven by Power (*M* = 2.88, SE = 0.32), which differed significantly from Joy (*M* = 1.72, SE = 0.33; *p* = 0.013), Tension (*M* = 2.00, SE = 0.26; *p* = 0.033), and Melancholy (*M* = 1.63, SE = 0.26; *p* = 0.003).

**Figure 5:**
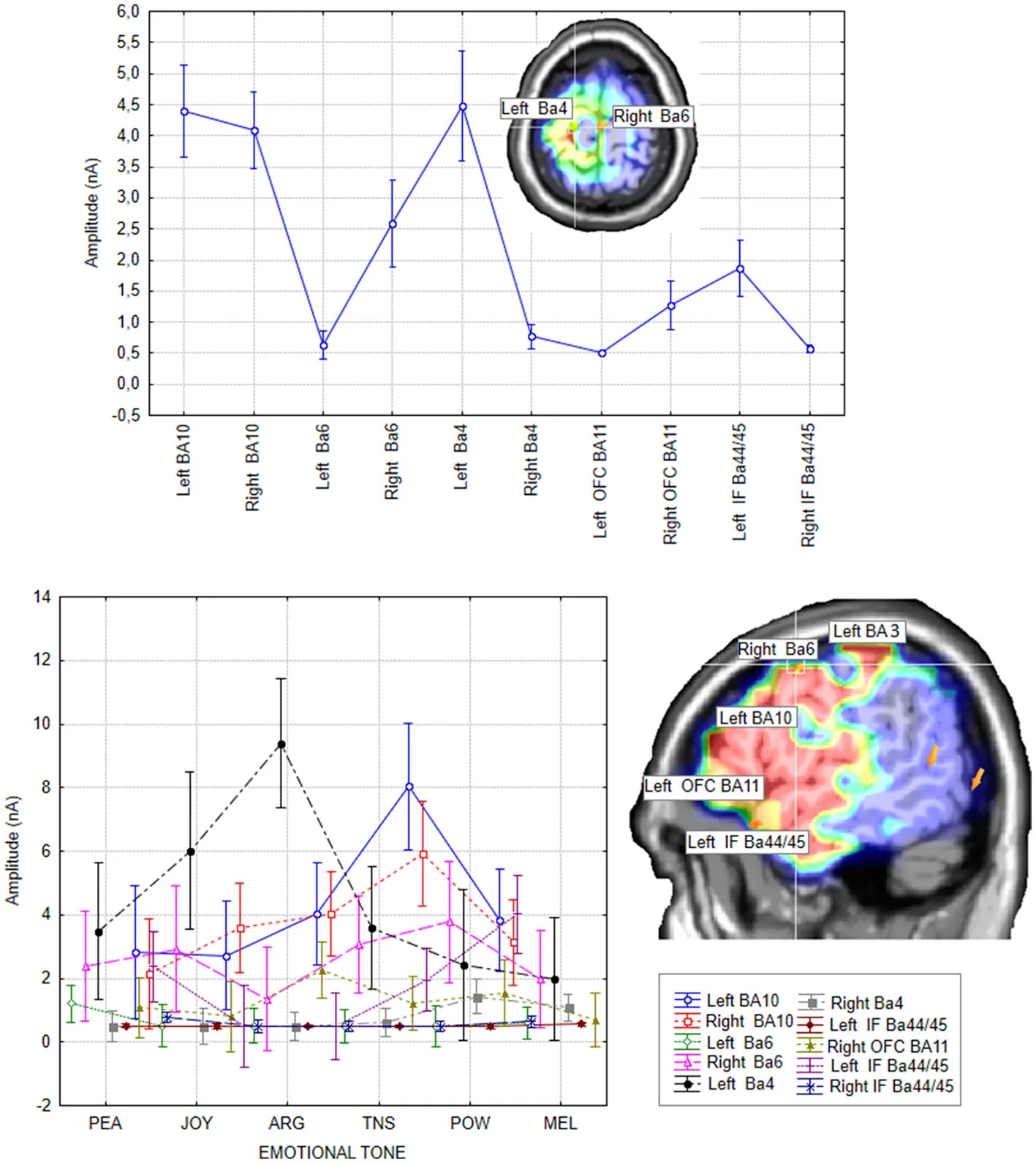
swLORETA-derived cortical source-current amplitudes according to repeated-measure ANOVA results. The upper panel shows mean amplitudes (nA) across bilateral frontal and motor regions, whereas the lower-left panel shows their variation across emotional categories (PEA, JOY, ARG, TNS, POW, and MEL). The swLORETA solutions in the upper and lower-right panels represent 2-s epochs recorded during the performance of Bach's *Contrapunctus VII* (Joy) and a Beethoven sonata (“Power” fragment), respectively. The maximum source-current amplitude displayed is 8 nA.

#### 3.2.3 Posterior region

The main effect of Area was significant [*F*_(10, 930)_ = 26.85, *p* < 0.0001], with left BA1/2/3 (primary somatosensory cortex; *M* = 4.55, SE = 0.48) emerging as the single most active area (see Figure 6), significantly exceeding all 10 other posterior regions (all *p* < 0.001), followed by left BA19 (*M* = 2.72, SE = 0.25) and left BA37 (*M* = 2.73, SE = 0.34), which did not differ from each other (*p* = 0.98) but both exceeded their right-hemisphere homologs. The Area × Emotion interaction was also significant [*F*_(50, 930)_ = 3.75, *p* < 0.001 (ε = 0.33, adjusted *p* < 0.001)]. Left BA1/2/3 was significantly modulated by emotional category, reaching its highest magnitude for Tension (*M* = 7.06, SE = 1.04) and Power (*M* = 6.51, SE = 1.29); notably, Melancholy was the only category in which the right-hemisphere homolog (right BA1/2/3, *M* = 3.96, SE = 0.65) also reached significance, suggesting a comparatively bilateral, strongly embodied somatosensory signature for this category. Left BA19 was significantly engaged during Peacefulness, Tension, and Power, and the left fusiform gyrus during Tension and Power.

**Figure 6:**
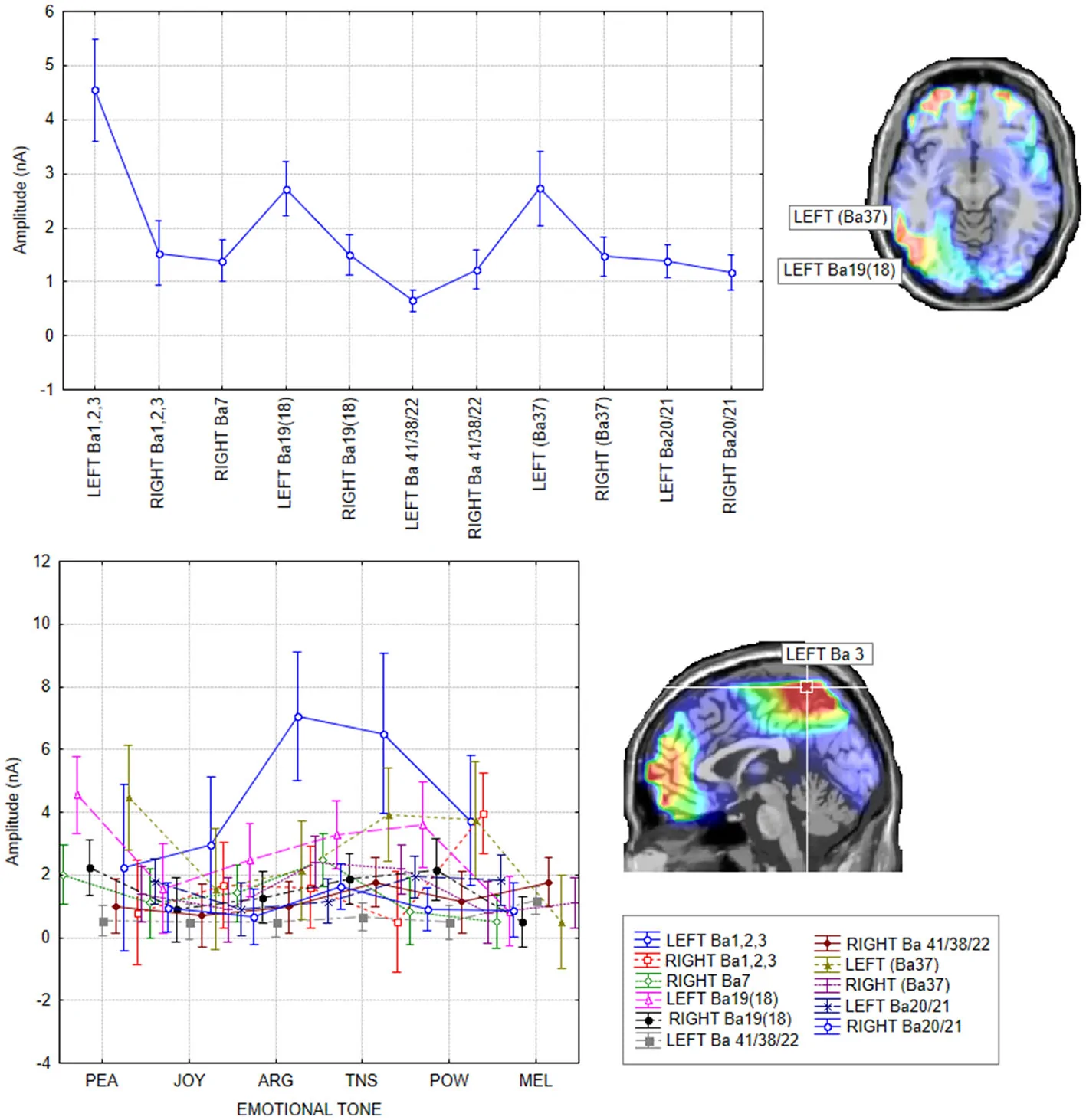
swLORETA-derived cortical source-current amplitudes according to repeated-measure ANOVA results. The upper-left panel shows mean amplitudes (nA) across selected bilateral posterior brain areas, whereas the lower-left panel shows their variation across emotional tones (PEA, JOY, ARG, TNS, POW, and MEL). The axial and sagittal swLORETA maps on the right represent a 2-s epoch recorded during the performance of a Beethoven sonata fragment classified as *Power*, with prominent activity in left BA37/BA19 (18) and left BA3, respectively. The maximum source-current amplitude displayed is 1.25 nA.

### 3.3 Correlations with structural performance parameters

#### 3.3.1 Frequency of activation

Pearson correlation analyses between regional activation frequency and objective performance parameters revealed a limited number of significant associations (Figure 7 top). Left thalamic activation frequency correlated positively with the number of left-hand notes per musical phrase (*r* = 0.894, *p* = 0.016), whereas right basal ganglia activation frequency correlated negatively with left-hand notes per phrase (*r* = −0.859, *p* = 0.028). At trend level, left insula activation frequency showed a negative association with gestures per second (*r* = −0.793, *p* = 0.060) and with left-hand notes per phrase (*r* = −0.774, *p* = 0.071), suggesting reduced insular engagement with increasing motor complexity. Left thalamic activation frequency also showed a positive trend with the overall number of notes per musical phrase (*r* = 0.760, *p* = 0.079). Pearson correlation analyses between anterior cortical activation frequency and objective performance parameters revealed a single significant association. Activation frequency of the left premotor cortex (BA6) correlated negatively with the number of left-hand notes per musical phrase (*r* = −0.829, *p* = 0.041). At trend level, left BA6 activation also showed negative associations with notes per musical phrase (*r* = −0.800, *p* = 0.056) and with acoustic energy (dBFS) (*r* = −0.759, *p* = 0.080). Pearson correlation analyses between posterior cortical activation frequency and objective performance parameters revealed no significant associations. At trend level, activation frequency of the left superior occipital gyrus/fusiform gyrus–middle occipital gyrus/cuneus/precuneus (BA19/18) showed positive associations with the overall number of notes per musical phrase (*r* = 0.799, *p* = 0.057) and with the number of right-hand notes per musical phrase (*r* = 0.752, *p* = 0.084).

**Figure 7:**
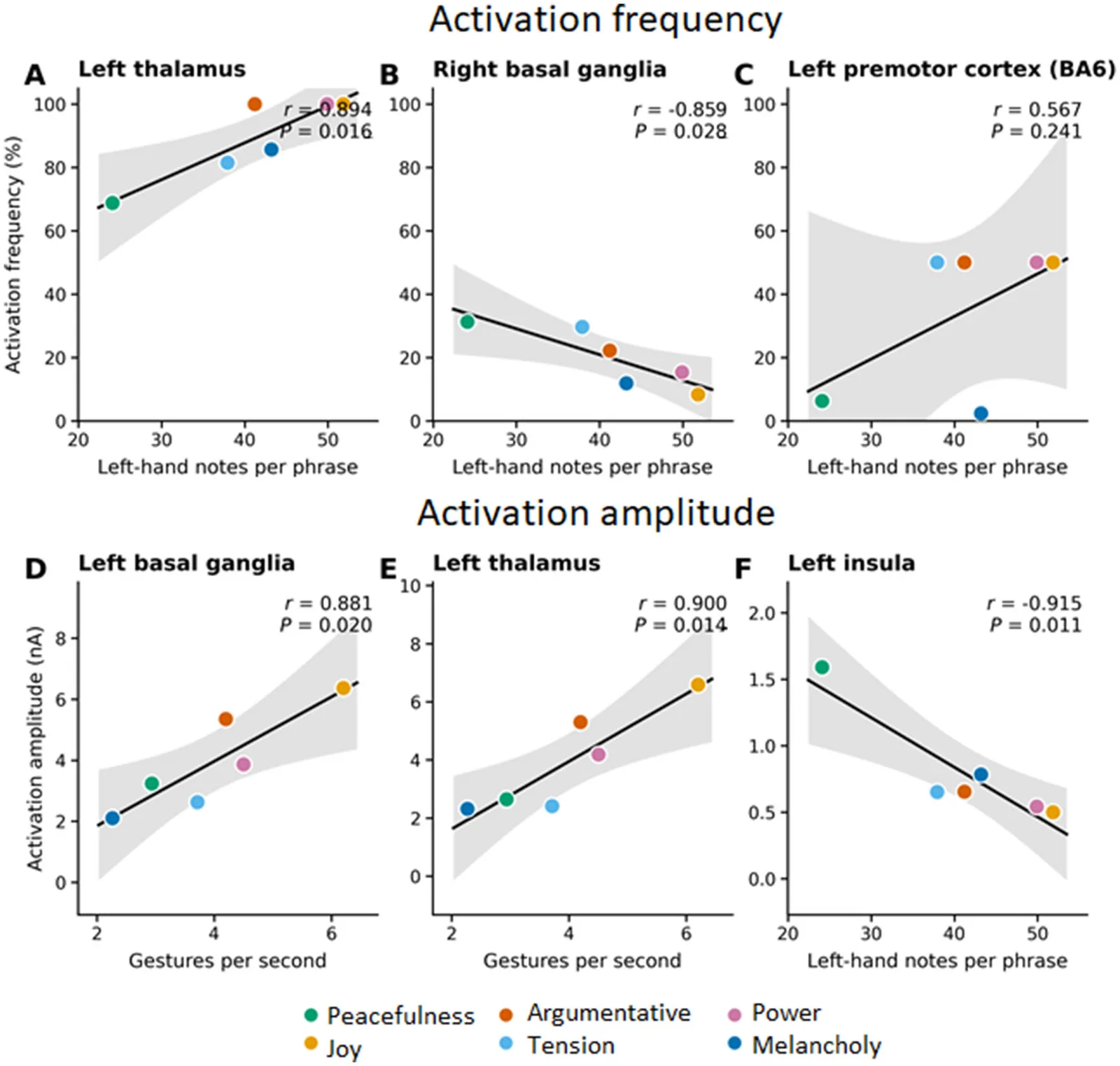
Selected Pearson correlation analyses between musical-performance features (as determined in Proverbio et al., 2026) and swLORETA-derived brain activity across emotional-tone conditions. Activation frequency in the left thalamus, right basal ganglia, and left premotor cortex (BA6) was correlated with the number of left-hand notes per phrase **(A–C)**. Activation amplitude in the left basal ganglia and left thalamus was correlated with gestures per second **(D, E)**, whereas left-insular amplitude was correlated with the number of left-hand notes per phrase **(F)**. Each point represents one emotional-tone condition. Solid lines show the linear fit, and shaded areas indicate 95% confidence intervals. Pearson's *r* coefficients and corresponding two-tailed *P* values are reported in each panel.

#### 3.3.2 Magnitude of activation

Pearson correlation analyses between the activation magnitude (nA) of subcortical regions and the objective performance parameters revealed several significant associations (see Figure 7 bottom). Activation of the left basal ganglia correlated positively with gestures per second (*r* = 0.881, *p* = 0.020), while left thalamic activation magnitude also showed a positive correlation with gestures per second (*r* = 0.901, *p* = 0.014), indicating greater recruitment of left subcortical motor regions during performances characterized by higher motor output. In contrast, left insular activation magnitude correlated negatively with the overall number of notes per musical phrase (*r* = −0.825, *p* = 0.043) and, more strongly, with the number of left-hand notes per musical phrase (*r* = −0.915, *p* = 0.011), suggesting reduced insular recruitment during performances with greater note density. Pearson correlation analyses between the activation magnitude (nA) of anterior cortical regions and the objective performance parameters revealed that magnitude of the left premotor cortex (BA6) was negatively correlated with the number of left-hand notes per musical phrase (*r* = −0.852, *p* = 0.031). No other significant correlations were found. None of the posterior cortical regions showed significant correlations with gestures per second, acoustic energy (dBFS), notes per musical phrase, or left- and right-hand note density.

## 4 Discussion

The present study used high-density EEG and swLORETA source reconstruction to characterize neural activity during emotionally categorized piano performance in a single elite pianist (case study). The results delineate a distributed and hierarchically organized performance system rather than an isolated subcortical motor axis. Across the six categories, a pre-dominantly left-lateralized cortico-striato-thalamo-sensorimotor architecture included the basal ganglia and thalamus, primary motor cortex (M1; BA4), primary somatosensory cortex (S1; BA1/2/3), premotor cortex (BA6), bilateral anterior prefrontal cortex (BA10), and posterior temporo-occipital regions. Within this shared architecture, emotional categories altered the relative contribution of motor implementation, sensory reafference, executive monitoring, and visuospatial or mnemonic processes, as indicated by the small-to-moderate association between anatomical district and emotion. The clearest affective contrast involved high subcortical magnitude during Joy and low subcortical magnitude, together with selective recruitment of right-hemisphere somatosensory and insular structures, during Melancholy; Power, Tension, Peacefulness, and Argumentative/Proactive showed additional, non-redundant cortical-subcortical configurations. Exploratory correlations further linked gesture rate and note density primarily to thalamic, basal-ganglia, insular, and premotor measures. Thus, expressive performance appears to arise from the dynamic coordination of action selection, motor command, tactile-proprioceptive feedback, prospective control, and affective modulation within a common expert-performance network.

### 4.1 A stable cortico-striato-thalamo-sensorimotor architecture for expert control

The most consistent result was the sustained recruitment of the basal ganglia and thalamus, pre-dominantly in the left hemisphere. These structures were active in approximately 85–100% of segments in every category and reached 100% occurrence in Argumentative/Proactive, Joy, and Power. Their pre-dominance was evident in both activation frequency and estimated source magnitude, and was accompanied by recurrent engagement of the left precentral gyrus (M1; BA4). This convergence is consistent with auditory-motor models of musical performance (Zatorre et al., 2007) and with evidence implicating cortico-basal-ganglia-thalamo-cortical circuits in action selection, temporal sequencing, motor fluency, and bimanual coordination (Parsons, 2005; Vaquero et al., 2016; Olszewska et al., 2024, 2025; Huang et al., 2024; Kuromiya et al., 2025). More broadly, the marked left-hemisphere pre-dominance observed in the present study is consistent with decades of research demonstrating that prolonged piano training induces experience-dependent functional and structural specialization throughout auditory-motor, cortico-striatal, and thalamo-cortical networks (Zatorre et al., 2007; Parsons, 2005; Vaquero et al., 2016; Merrick et al., 2023; Olszewska et al.,2024,2025). Rather than reflecting a generalized hemispheric asymmetry, this left-dominant pattern may reflect a highly automatized sensorimotor organization in this extensively trained pianist, enabling rapid sequencing, precise bimanual coordination, and efficient translation of expressive intentions into skilled motor output. Nevertheless, given the single-case design of the present study, this interpretation should be regarded as consistent with, rather than demonstrative of experience-dependent neuroplasticity. Importantly, however, the subcortical findings should not be interpreted in isolation. In the present performance, thalamic and striatal recruitment was embedded in a broader cortical loop in which BA4 implemented the selected motor programs, somatosensory areas supplied continuous information about touch and limb position, premotor regions organized sequential and bimanual actions, and prefrontal regions maintained the higher-order interpretive plan.

The cortical results provide a more complete account of this performance architecture. Left BA4, together with bilateral BA10, formed the highest-magnitude anterior cluster, indicating that the neural organization of expert playing involved both direct motor implementation and sustained supraordinate control. BA4 is the final cortical stage through which temporally organized action plans are translated into the precisely graded finger and hand movements required to shape articulation, timing, and dynamics (Yokoi et al., 2018). At the same time, the left primary somatosensory cortex (BA1/2/3) was the single most active posterior region and significantly exceeded all other posterior areas in overall magnitude. Its strong recruitment suggests that, in the present performance, piano playing involved not only the issuing of motor commands but also the continuous processing of tactile and proprioceptive reafference from the fingers, hands, and upper limbs. This sensory information is essential for monitoring key contact, force, position, and movement consequences and for updating ongoing actions with minimal delay. The particularly high BA1/2/3 magnitude during Tension and Power further suggests that passages requiring forceful, dense, or precisely controlled gestures place increased demands on embodied sensorimotor monitoring. Recurrent right premotor activity may contribute to left-hand control, anticipatory organization, and coordination of the two hands, whereas the near-ubiquitous bilateral recruitment of BA10 may support prospective monitoring, working memory, sustained attention, and maintenance of the interpretive goal throughout a long, self-paced performance. Finally, activity in BA19, BA37, the cuneus, precuneus, and fusiform cortex indicates that the performing brain also recruits posterior systems potentially related to visuospatial organization, learned score or keyboard representations, musical imagery, and access to long-term structural knowledge. The resulting picture is therefore one of an integrated perception-action cycle, in which cortical motor output, somatosensory feedback, subcortical sequencing, and executive supervision operate continuously and in parallel.

### 4.2 Emotion-related modulation of the shared performance network

Joy elicited the highest overall subcortical magnitude and was significantly greater than Tension and Melancholy, with particularly pronounced peaks in the left thalamus and left basal ganglia. This profile indicates maximal engagement of the left subcortical motor loop during this positive, high-arousal category. Joy also involved recurrent left BA4 and right postcentral recruitment, suggesting that its neural profile was not exclusively subcortical but combined motor implementation with sensory monitoring of a rapid and highly articulated performance. Such engagement may reflect the joint requirements of fluent gesture production, temporal precision, and positive expressive activation; however, because emotional category covaried with objective motor and acoustic parameters (see Table 1), a specifically reward- or valence-based interpretation remains tentative. Melancholy showed the lowest subcortical magnitude, differing significantly from Joy, Argumentative/Proactive, and Power. At the same time, it selectively recruited right-hemisphere structures, including right BA10, the right primary somatosensory cortex, and the right insula/claustrum, while preserving strong left basal-ganglia and thalamic involvement. The simultaneous recruitment of right S1 and insular regions points to a comparatively bilateral and embodied configuration, potentially emphasizing bodily-state monitoring and the sensory-affective qualities of touch rather than motor output alone. This pattern is broadly compatible with models linking negative or withdrawal-related affect to greater right-hemisphere participation (Henriques and Davidson, 1990, 1991; Sutton and Davidson, 1997; Gainotti, 2019, 2021) and with right-sided responses to sad music in listeners (Mitterschiffthaler et al., 2007; Proverbio and Piotti, 2021; Proverbio, 2025). Importantly, the present data indicate selective rightward additions to a persistent left motor core, rather than a global reversal of hemispheric dominance.

The remaining categories also displayed informative profiles that argue against reducing the findings to a Joy-Melancholy dichotomy or to a unitary arousal-related increase in activation. Power produced the highest mean activation in the anterior macro-region, exceeding Joy, Tension, and Melancholy, and was uniquely associated with increased right cerebellar and right thalamic magnitude together with a renewed left thalamic peak. Combined with high left BA4 and BA1/2/3 recruitment, this configuration suggests coordinated engagement of motor output, sensory feedback, executive supervision, and cerebellar error correction during forceful and acoustically intense performance. Tension was characterized by bilateral BA10 recruitment and the highest left primary somatosensory magnitude, accompanied by engagement of left BA19 and the fusiform cortex. In addition, tension-related excerpts preferentially recruited the left supramarginal gyrus (BA40) and right superior parietal cortex (BA7), supporting multisensory integration, proprioceptive–visuospatial updating, and the control of effortful, tightly regulated movements. Its profile therefore appears to emphasize continuous monitoring of both the bodily consequences of movement and the spatial or structural organization of the passage. Peacefulness combined comparatively moderate motor recruitment with prominent left temporo-occipital activity involving BA19, fusiform cortex, cuneus, and precuneus; in a memorized performance, this pattern may reflect greater reliance on internally generated visuospatial representations, musical imagery, and retrieval of learned structural configurations. Argumentative combined strong left BA4 and left subcortical recruitment with a right-prefrontal profile involving BA10 and BA11, consistent with forceful motor implementation under sustained goal-directed monitoring. These dissociations show that emotional categories reorganized different stages of the performance loop: Joy preferentially amplified thalamo-striatal engagement, Power recruited an extended motor-sensory-cerebellar configuration, Tension maximized somatosensory monitoring, Peacefulness emphasized posterior representational systems, and Melancholy added right-sided sensory-affective nodes. The modest overall effect size of the Area by Emotion association (Cramer's V = 0.22) is therefore theoretically informative: emotional categories altered the relative weighting and coupling of nodes within a largely shared network rather than being encoded by anatomically discrete circuits. This interpretation is compatible with constructionist accounts of musical emotion (Cespedes-Guevara and Eerola, 2018). It should be emphasized that the emotional categories refer not to emotions necessarily experienced by the pianist, but to those perceived by listeners. These perceptual judgments arise from the interaction between the performer's expressive interpretation and the structural characteristics of the musical piece. Both factors are likely to shape the way in which the pianist plans, controls, and executes movements, while any emotional states actually experienced during performance may additionally recruit affective and interoceptive regions, such as the insular cortex, the limbic system, or the temporal and orbitofrontal cortices (Koelsch, 2014).

### 4.3 Motor complexity and neurofunctional engagement

The correlational analyses provided convergent, although exploratory, evidence that source recruitment varied with objective properties of the performance. At the level of activation magnitude, left basal-ganglia and left thalamic activity correlated positively with gestures per second, indicating stronger subcortical recruitment in categories characterized by greater motor output. At the level of activation frequency, the left thalamus correlated positively with the number of left-hand notes per phrase, whereas right basal-ganglia frequency correlated negatively with the same parameter. These complementary associations may reflect a redistribution of activity within the bilateral subcortical motor system as the structural and manual demands of the passages change, rather than a uniform increase across all motor regions. They are consistent with the role of basal-ganglia-thalamic loops in movement selection, sequencing, sensory gating, and automaticity (Alexander and Crutcher, 1990; Middleton and Strick, 2000; Parsons, 2005; Vaquero et al., 2016). Within the broader network described above, these subcortical changes can be understood as modulating the flow of information between premotor planning, M1 output, and somatosensory feedback as the required gesture rate increases.

The left insula and left premotor cortex showed an inverse pattern. Left insular magnitude decreased as the total number of notes and the number of left-hand notes increased; activation-frequency associations with gesture rate and left-hand note density were directionally similar but did not reach conventional significance. Left BA6 frequency and magnitude were both negatively associated with left-hand note density, with additional non-significant trends involving total note count and acoustic intensity. These findings may indicate that highly practiced, note-dense passages rely less on sustained premotor planning and interoceptive monitoring and more on automatized subcortical control and rapidly updated sensorimotor loops. Indeed, the pieces were highly overlearned, as the musician had performed them repeatedly in concert and recorded them on CD. This interpretation is consistent with accounts of the insula as an interface between interoception, salience, and goal-directed control (Craig, 2009; Menon and Uddin, 2010) and with models of motor skill acquisition in which premotor demands change as action sequences become automatized (Engel et al., 1997; Hikosaka et al., 2002). Another possibility is that note-dense passages were relatively poor in emotional pathos and therefore elicited less insular engagement. Consistently, the melancholic excerpts in the present dataset tended (Gu et al., 2013) to contain fewer notes, paralleling previous evidence that sadness-related musical excerpts are characterized by low note density (Shen et al., 2018). Nevertheless, the data are correlational and do not demonstrate a functional transfer from cortical to subcortical systems; BA6, BA4, S1, thalamic, and striatal contributions are more plausibly viewed as interacting components of a continuously recurrent control system.

No posterior cortical region showed a significant linear correlation with gesture rate, acoustic energy, total note count, or hand-specific note density. Likewise, the robust recruitment of left BA4 was not captured by the reported performance-parameter correlations. This absence is important because it indicates that the prominent M1 and S1 responses visible across categories cannot be reduced to the simple amount of movement or to note count alone. Primary motor and somatosensory activity may constitute task-essential components that remain continuously engaged, while their category-specific magnitude reflects non-linear or unmeasured aspects of performance, such as fingering configuration, articulation, force scaling, intermanual coordination, expressive timing, tactile prediction, and sensory prediction error. Thus, the pronounced left S1 activation during Tension and Power, the bilateral somatosensory involvement during Melancholy, and the temporo-occipital engagement observed during Peacefulness, Tension, and Power likely represent qualitatively different modes of sensorimotor and representational control. Moreover, acoustic intensity itself was not significantly associated with any regional measure. The most defensible conclusion is therefore that gestural rate and note density account for part of the variation in subcortical, insular, and premotor recruitment, whereas M1, S1, and posterior cortical profiles capture additional dimensions of expert control that were not indexed by the available summary parameters.

### 4.4 Limitations, interpretation, and future directions

Several limitations constrain the interpretation of these findings. Most importantly, this was a single-case study of one exceptionally trained, right-handed male pianist. The large number of analyzed segments increases within-performance resolution but does not increase the biological sample size, and the segments were nested within musical phrases and within one continuous performance. Although temporal-spacing and selection criteria were used to reduce local autocorrelation, observations from a single performance cannot be assumed to be fully independent. Replication across pianists, instruments, expertise levels, handedness, and repertoires is therefore necessary before the observed lateralization patterns can be generalized.

A second limitation concerns the interpretation of the emotion categories. The labels were validated through listener judgments and should be understood as externally recognized expressive profiles, not as direct measures of the pianist's subjective emotional experience or moment-to-moment expressive intention. In addition, emotional category was intrinsically coupled to tempo, mode, note density, gesture rate, manual distribution, acoustic energy, composer, and passage structure. The naturalistic design is a major strength for ecological validity, but it prevents a clean separation of affective modulation from the motor-acoustic properties through which emotion is communicated. Future studies should combine listener ratings with performer self-report and continuous dimensional measures of valence and arousal, while comparing passages that are matched as closely as possible for tempo, note density, dynamics, and manual demands.

A third limitation concerns source localization and statistical inference. swLORETA provides model-based estimates of intracranial generators from scalp EEG and cannot offer the spatial precision of direct recordings or high-field functional imaging, particularly for deep structures such as the thalamus and basal ganglia. Subcortical findings should therefore be interpreted as convergent source estimates rather than direct measurements of local neural activity. In addition, the performance-parameter correlations were calculated across six emotional-category summaries and involved multiple regional tests. The resulting coefficients may be unstable, are vulnerable to influential category means, and should be regarded as hypothesis-generating. Confirmatory studies should use larger performer samples, multilevel models that preserve the nesting of segments within phrases and individuals, preregistered contrasts, and correction for multiple comparisons. On a final note, because the 99 EEG segments were obtained from a single continuous performance and were nested within musical phrases, the observations cannot be regarded as fully independent. Consequently, the inferential statistics reported here may underestimate uncertainty and thereby potentially overstate statistical significance. These analyses should therefore be interpreted cautiously as exploratory within-performance analyses rather than as population-level evidence.

Despite these constraints, the present findings suggest that, in this case, emotionally expressive piano performance engaged a distributed cortical-subcortical architecture rather than a single motor pathway. A stable left-dominant fronto-striato-thalamic scaffold operates together with M1, primary somatosensory cortex, premotor and prefrontal regions, cerebellum, and posterior temporo-occipital systems. Within this architecture, basal ganglia and thalamus support selection, sequencing, and motor fluency; BA4 implements the detailed motor command; BA1/2/3 provides continuous tactile-proprioceptive information; premotor and cerebellar systems contribute anticipatory organization and error correction; BA10 maintains the interpretive goal; and posterior regions may support visuospatial, mnemonic, and imaginal representations. Emotional expression appears to dynamically reweight these interacting components, producing category-specific configurations without abolishing the common performance network. Combining high-density EEG with motion capture, finger-force measures, electromyography, autonomic indices, performer reports, and complementary imaging modalities will be important for determining which components reflect affective intention, movement execution, sensory feedback, learned automaticity, and their moment-to-moment interaction. Potential applications to performance training and neurorehabilitation are promising, but remain to be tested in larger and clinically relevant samples.

## 5 Conclusions

The present findings suggest that, within this naturalistic, concert-like setting, piano performance was supported by a highly specialized neural architecture, anchored in a stable left fronto-striato-thalamic axis and modulated, in its peripheral configurations, by the affective quality of the interpretive experience and the motor complexity of the performed material. By reconstructing, for the first time in an elite performer, the cortical and subcortical generators associated with distinct emotional categories during live performance, this study extends the neuroscience of musical emotion from the listener's to the performer's perspective and provides a quantitative bridge between structural performance parameters and the neurofunctional architecture of musical expressivity.

## Data Availability

The original contributions presented in the study are included in the article/Supplementary material, further inquiries can be directed to the corresponding author.
