# Supplementary material for: Neural dynamics of emotionally expressive piano performance: a single-case study
Source: Front Cognit. 2026 Oct 6;5:1945714. doi: 10.3389/fcogn.2026.1945714 (PMC13680138; doi:10.3389/fcogn.2026.1945714)
Supplement: Supplementary file 1 [file Table_1.docx]

**Supplementary file**

| **Table 1.** Brain-region equivalence table used to group regions belonging to the same Brodmann area. | | |
| --- | --- | --- |
| **Macro-Category** | **Gyrus or brain source** | **BA swLORETA** |
| Superior/Middle Frontal Gyrus | Superior Frontal Gyrus, Medial Frontal Gyrus, Middle Frontal Gyrus | 10 |
| Basal Ganglia | Lentiform Nucleus, Putamen, Caudate (Body & Head) | / |
| Thalamus | Thalamus (Lateral Posterior Nucleus, Medial Dorsal Nucleus) | / |
| Insula & Claustrum | Claustrum, Insula | 13 |
| IPL/Supramarginal Gyrus | Inferior Parietal Lobule, Supramarginal Gyrus (T) | 40 |
| MOG/IT/FG | Inferior Temporal Gyrus (T), Fusiform Gyrus (T/O), Middle Occipital Gyrus (O) | 37 |
| ITG/FG | Inferior Temporal Gyrus, Fusiform Gyrus | 20 |
| FG/MOG/Cuneus/Precuneus | Fusiform Gyrus (T), Cuneus (O), Middle Occipital Gyrus (O), Precuneus (P), Superior Occipital Gyrus (O) | 19 |
| IOG/LG/MOG | Inferior Occipital Gyrus (O), Lingual Gyrus (O), Middle Occipital Gyrus (O), Cuneus (O) | 18 |
| MFG | Medial Frontal Gyrus, Middle Frontal Gyrus | 11 |
| Premotor Cortex | Medial Frontal Gyrus, Superior Frontal Gyrus, Precentral Gyrus (F) | 6 |
| Cerebellum | Posterior Lobe (Declive), Cereb, nan | / |

**Table 2**. Anatomical subdivision of swLORETA-derived regions into three macroregional subgroups (anterior, posterior, and subcortical) for subsequent statistical analyses.

| **Macroregion** | **Anatomical Region** | **BA** | **Hemisphere** |
| --- | --- | --- | --- |
| ANTERIOR | Superior/Middle Frontal Gyrus | BA10 | Left |
| ANTERIOR | Superior/Middle Frontal Gyrus | BA10 | Right |
| ANTERIOR | Premotor Cortex | BA6 | Left |
| ANTERIOR | Premotor Cortex | BA6 | Right |
| ANTERIOR | Precentral Gyrus | BA4 | Left |
| ANTERIOR | Precentral Gyrus | BA4 | Right |
| ANTERIOR | Medial Frontal Gyrus (OFC) | BA11 | Left |
| ANTERIOR | Medial Frontal Gyrus (OFC) | BA11 | Right |
| ANTERIOR | Inferior Frontal Gyrus | BA44/45 | Left |
| ANTERIOR | Inferior Frontal Gyrus | BA44/45 | Right |
| POSTERIOR | Postcentral Gyrus | BA1, 2, 3 | Left |
| POSTERIOR | Postcentral Gyrus | BA1, 2, 3 | Right |
| POSTERIOR | Superior Parietal Lobule | BA7 | Left |
| POSTERIOR | Superior Parietal Lobule | BA7 | Right |
| POSTERIOR | Superior Occipital Gyrus, Fusiform Gyrus/Middle Occipital Gyrus, Cuneus/Precuneus | BA19 (18) | Left |
| POSTERIOR | Superior Occipital Gyrus, Fusiform Gyrus/Middle Occipital Gyrus, Cuneus/Precuneus | BA19 (18) | Right |
| POSTERIOR | Auditory Cortex | BA41/38/22 | Left |
| POSTERIOR | Auditory Cortex | BA41/38/22 | Right |
| POSTERIOR | Fusiform Gyrus | BA37 | Left |
| POSTERIOR | Fusiform Gyrus | BA37 | Right |
| POSTERIOR | Middle/Inferior Temporal Cortex | BA20/21 | Left |
| POSTERIOR | Middle/Inferior Temporal Cortex | BA20/21 | Right |
| SUBCORTICAL | Basal Ganglia (Caudate/Putamen) | — | Left |
| SUBCORTICAL | Basal Ganglia (Caudate/Putamen) | — | Right |
| SUBCORTICAL | Cerebellum | — | Left |
| SUBCORTICAL | Cerebellum | — | Right |
| SUBCORTICAL | Thalamus (Lateral Posterior/Mediodorsal Nuclei) | — | Left |
| SUBCORTICAL | Thalamus (Lateral Posterior/Mediodorsal Nuclei) | — | Right |
| SUBCORTICAL | Uncus | BA36/28 | Left |
| SUBCORTICAL | Uncus | BA36/28 | Right |
| SUBCORTICAL | Insula/Claustrum | BA13 | Left |
| SUBCORTICAL | Insula/Claustrum | BA13 | Right |

| **Table 3.** Chi-square comparisons of activation frequencies across emotional conditions and brain regions.  Chi-square (χ²) statistics are reported for each cerebral region and hemisphere across the PEA, JOY, ARG, TNS, POW, and MEL conditions. Higher χ² values indicate a greater discrepancy between the observed and expected activation frequencies, whereas values approaching zero indicate minimal deviation from the expected distribution. BA denotes Brodmann area; FG, fusiform gyrus; MOG, middle occipital gyrus; OFC, orbitofrontal cortex; Sup. occ., superior occipital; and Sup. parietal, superior parietal; Hem., hemisphere. | | | | | | | |
| --- | --- | --- | --- | --- | --- | --- | --- |
| **Brain area** | **Hem.** | **PEA** | **JOY** | **ARG** | **TNS** | **POW** | **MEL** |
| Basal ganglia (caudate/putamen) | Left | 0.033 | 15.515 | 10.305 | 0.002 | 0.500 | 0.694 |
| Basal ganglia (caudate/putamen) | Right | 0.946 | 0.310 | 0.393 | 0.016 | 0.490 | 0.281 |
| Cerebellum | Left | 0.000 | 0.187 | 0.003 | 0.015 | 0.001 | 0.032 |
| Cerebellum | Right | 0.012 | 23.483 | 0.615 | 0.091 | 13.792 | 0.278 |
| Thalamus (lateral posterior/medial dorsal) | Left | 10.067 | 20.816 | 15.734 | 0.003 | 0.255 | 0.371 |
| Thalamus (lateral posterior/medial dorsal) | Right | 0.032 | 15.643 | 32.209 | 0.022 | 11.998 | 17.923 |
| Uncus Ba36/28 | Left | 0.147 | 0.004 | 0.095 | 0.242 | 15.141 | 0.005 |
| Uncus Ba36/28 | Right | 0.147 | 0.649 | 19.618 | 0.242 | 0.150 | 0.304 |
| Insula/Claustrum Ba 13 | Left | 0.494 | 0.671 | 0.936 | 0.166 | 11.884 | 17.229 |
| Insula/Claustrum Ba 13 | Right | 14.781 | 31.691 | 12.017 | 0.491 | 22.582 | 23.314 |
| Superior/middle Frontal gyrus BA10 | Left | 0.008 | 0.002 | 0.338 | 0.012 | 0.012 | 0.101 |
| Superior/middle Frontal gyrus BA10 | Right | 0.050 | 0.977 | 0.493 | 0.009 | 0.947 | 0.068 |
| Premotor cortex Ba6 | Left | 0.146 | 0.050 | 0.022 | 0.107 | 0.051 | 0.024 |
| Premotor cortex Ba6 | Right | 0.556 | 0.012 | 21.353 | 13.679 | 0.161 | 0.033 |
| Precentral gyrus Ba4 | Left | 0.338 | 11.852 | 84.321 | 0.841 | 3.792 | 32.092 |
| Precentral gyrus Ba4 | Right | 0.934 | 0.219 | 0.773 | 0.003 | 0.792 | 1.365 |
| Medial frontal gyrus OFC BA11 | Left | 0.016 | 0.102 | 0.003 | 0.053 | 0.017 | 0.084 |
| Medial frontal gyrus OFC BA11 | Right | 17.634 | 0.048 | 95.442 | 0.894 | 0.194 | 43.704 |
| Inferior frontal gyrus Ba44/45 | Left | 0.149 | 18.278 | 36.772 | 0.147 | 28.658 | 10.335 |
| Inferior frontal gyrus Ba44/45 | Right | 38.021 | 0.004 | 0.195 | 0.397 | 0.001 | 0.479 |
| Postcentral gyrus Ba1,2,3 | Left | 0.002 | 0.154 | 36.531 | 0.484 | 0.555 | 17.492 |
| Postcentral gyrus Ba1,2,3 | Right | 18.677 | 134.962 | 0.961 | 0.320 | 18.863 | 0.055 |
| Superior parietal lobule Ba7 | Left | 0.000 | 0.187 | 0.003 | 0.015 | 0.001 | 0.032 |
| Sup. parietal lobule Ba7 | Right | 0.732 | 0.961 | 21.632 | 0.276 | 60.563 | 25.158 |
| Sup. occ. gyrus, FG/MOG/Cuneus/Precuneus Ba19(18) | Left | 0.790 | 49.873 | 17.974 | 11.697 | 0.062 | 0.967 |
| Sup. occ. gyrus, FG/MOG/Cuneus/Precuneus Ba19(18) | Right | 41.727 | 19.161 | 38.296 | 0.003 | 13.401 | 0.061 |
| Auditory cortex Ba 41/38/22 | Left | 0.099 | 22.113 | 0.436 | 0.740 | 0.102 | 0.967 |
| Auditory cortex Ba 41/38/22 | Right | 12.089 | 0.035 | 25.712 | 0.181 | 0.021 | 0.078 |
| Fusiform gyrus (Ba37) | Left | 33.583 | 32.555 | 61.241 | 0.001 | 0.151 | 17.492 |
| Fusiform gyrus (Ba37) | Right | 0.469 | 0.058 | 62.228 | 0.254 | 14.341 | 37.466 |
| Middle inf. Temporal cortex Ba20/21 | Left | 0.549 | 37.508 | 60.624 | 0.003 | 19.447 | 22.429 |
| Middle inf. Temporal cortex Ba20/21 | Right | 12.089 | 60.673 | 132.598 | 3.147 | 25.294 | 0.342 |

| **Table 4.**  Emotional categories used for classification of 110 musical excerpts (lasting ~11 sec). While the structural differences were objective, the emotional label reflected a psychological sensation (taken form Proverbio & Milovanović, 2026). | | | | |
| --- | --- | --- | --- | --- |
| **Emotional valence** | **Emotional feeling** | **Key** | **Tempo** | **Energy/Arousal** |
| Positive | Peacefulness (PEA) | Major | Slow | Little |
| Positive | Joy (JOY) | Major | Fast | Considerable |
| Negative | Melancholy (MEL) | Minor | Slow | Little |
| Negative | Argumentative (ARG) | Minor | Fast | Some |
| Negative | Tension (TNS) | Minor | — | Considerable |
| — | Power (POW) | — | — | Strong |
